# Data-driven identification of subtypes of intimate partner violence

**DOI:** 10.1038/s41598-021-85947-3

**Published:** 2021-03-24

**Authors:** Ahmet Mert Hacıaliefendioğlu, Serhan Yılmaz, Douglas Smith, Jason Whiting, Mehmet Koyutürk, Günnur Karakurt

**Affiliations:** 1 Department of Computer and Data Sciences, Case Western Reserve University, Cleveland, OH USA; 2 Center for Proteomics and Bioinformatics, Case Western Reserve University, Cleveland, OH USA; 3 Department of Applied and Professional Studies, Texas Tech University, Lubbock, TX USA; 4 Brigham Young University, Provo, Utah USA; 5 Department of Psychiatry, Case Western Reserve University, Cleveland, OH 44106 USA; 6 University Hospitals Cleveland Medical Center, Cleveland, OH USA

**Keywords:** Computational biology and bioinformatics, Psychology, Mathematics and computing

## Abstract

Intimate partner violence (IPV) is a complex problem with multiple layers of heterogeneity. We took a data-driven approach to characterize this heterogeneity. We integrated data from different studies, representing 640 individuals from various backgrounds. We used hierarchical clustering to systematically group cases in terms of their similarities according to violence variables. Results suggested that the cases can be clustered into 12 hierarchically organized subgroups, with verbal abuse and negotiation being the main discriminatory factors at higher levels. The presence of physical assault, injury, and sexual coercion was discriminative at lower levels of the hierarchy. Subgroups also exhibited significant differences in terms of relationship dynamics and individual factors. This study represents an attempt toward using integrative data analysis to understand the etiology of violence. These results can be useful in informing treatment efforts. The integrative data analysis framework we develop can also be applied to various other problems.

## Introduction

Intimate partner violence (IPV) is a highly complex and multi-faceted problem. It includes the broad range of acts that can be physical, emotional, and sexual in nature^1–3^. IPV includes intentions to control, subdue, punish, or isolate someone by utilizing tactics such as manipulation, humiliation, or fear^4^ along with physically forced or coerced sexual acts or attempts to obtain a sexual act through pressure^5^. The presence of physical and non-physical forms of violence can be highly related^1^.

Violent behaviors vary by severity, mutuality, and the generality of violence^2,6,7^. Prior research analyzing the psycho-physiological data also indicate typological differences between impulsive and premeditated aggression^8^. The factors that underlie violence are also multi-faceted due to differences in perpetrators, victims, and relationship context^9^. Witnessing violence during childhood, substance abuse issues, gender role socialization, personality traits, stress, affect regulation, attachment security and psychopathology are all significant contributors to IPV^8,10,11^.

All of these factors are critical in understanding the subtypes of violence for guiding treatment efforts. Recently, subgroups of violent offenders were identified based on anger experience, expression, and control, and referred to treatment accordingly^12^. It was also shown that consideration of subtypes added differential benefit to treatment outcome^12^. Similarly, a subtype of violent offenders that had a higher risk for greater criminogenic needs and higher rates of sexual violence was identified, and this information was used to predict within-treatment changes in a violence reduction program^6^. The frequency and severity of violence, the generality of violence, and personality traits were also utilized to identify clinically-relevant subtypes of violence indicating the effectiveness of treatment is highly dependent on the subtypes of violence^9^.

In this study, we aimed to identify subgroups of perpetrators through a multi-dimensional conceptualization of violence. We developed a data-driven approach and integrated datasets from multiple sources to identify subgroups using an unsupervised, i.e., data-driven, clustering approach. We hypothesized that clustering of cases in integrated datasets would lead to data-driven discovery of subgroups that share common characteristics. Subgroup analysis in a hierarchical clustering context was also expected to provide a systematic understanding of the distinctions between subgroups in terms of the existence, severity, and types of violence. Furthermore, we used enrichment analyses to project these subgroups to personal, social, and relationship variables that are not considered in clustering.

## Methods

Our objective in this study was to identify subgroups of relationships/people in a relationship that represent subgroups of IPV. For this purpose, we integrated multiple sources data that includes measurements of conflict in relationships. The datasets included individuals who did not experience IPV as well as perpetrators and victims/survivors, but the datasets do not designate between perpetrators and victims of IPV. We aimed to cluster all participants for which conflict data was available (without making the presence of IPV a criterion for inclusion), so that we could provide a data-driven, unbiased picture of the distribution of conflict and violence across the population.

**Figure 1:**
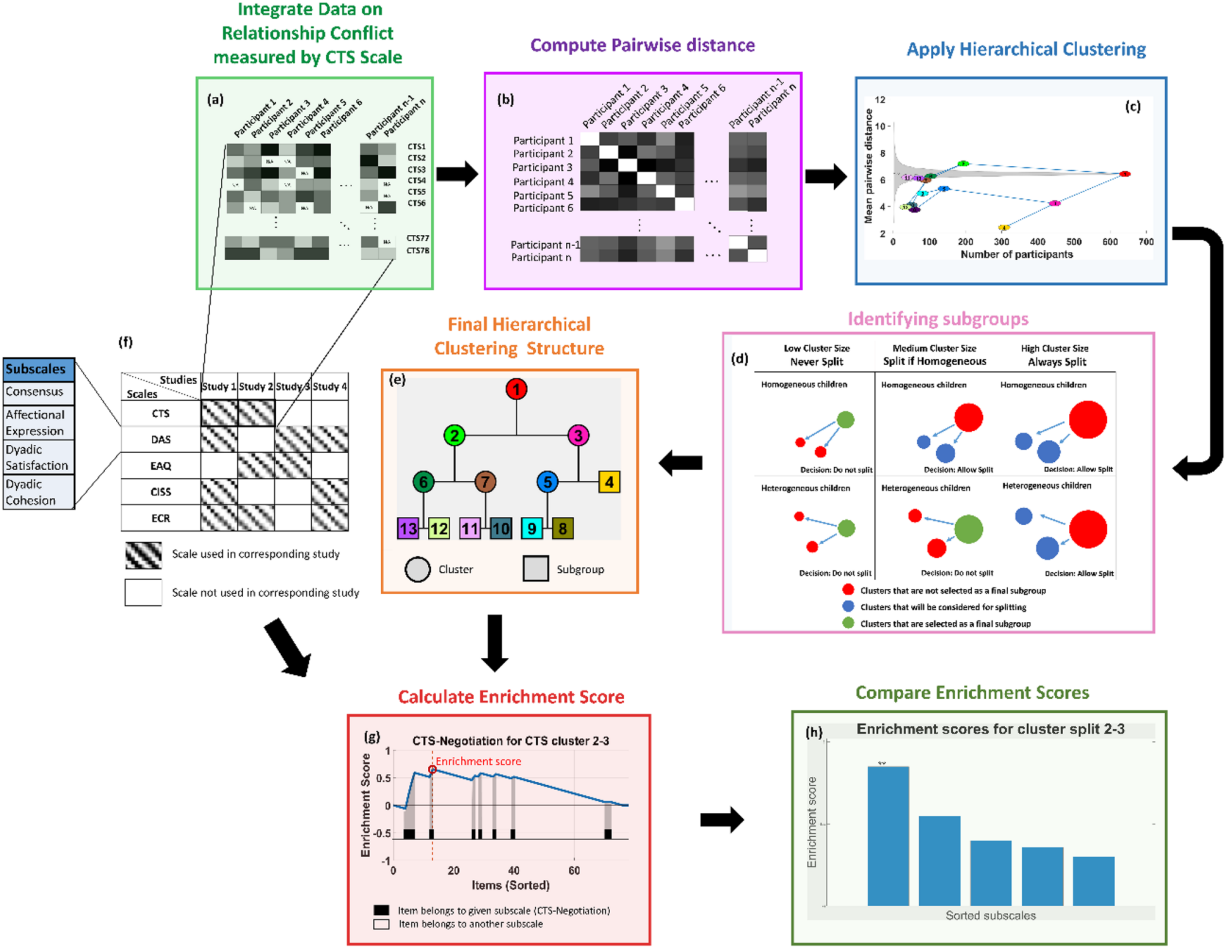
The workflow of our integrative data analysis framework for the identification and annotation of intimate partner violence (IPV) subgroups. (**a**) We first integrated data from different studies that employ Conflicts Tactics Scale (CTS), (**b**) we computed pairwise distances between all pairs of participants by accounting for missing data to enable incorporation of partial data in some studies, (**c**) we performed agglomerative hierarchical clustering on the resulting distance matrix, (**d**) we used cluster size and cluster homogeneity to decide on cluster boundaries and (**e**) identify subgroups, (**f**) we assessed the difference in the distribution of each CTS item on each cluster split and perform enrichment analysis to identify CTS subscales that exhibit a significant difference with respect to each cluster split, (**g**) we referred back to the integrated studies to (**h**) project the CTS subgroups to other scales measuring personal factors, relationship context, and mental health, with a view to identifying variables that exhibit a significant difference between different subgroups.

Our approach called for a unique analytical framework due to the heterogeneous nature of violent behaviors and methodological challenges in integrating disparate data sources. To overcome these challenges, we applied multiple techniques. First, we developed a distance measure to assess the dissimilarity between pairs of participants in the presence of missing data. Then, we visualized the hierarchical clustering of participants in a space that represents the trade-off between cluster size and homogeneity. Finally, we applied enrichment analysis to score and rank subscales measuring a broad range of psychological constructs, based on the collective ability of their items in distinguishing subgroups. Our methodology’s detailed workflow is shown in Fig. 1.

### Data integration and description of data

We combined data from three different sources to capture the diversity and heterogeneity of the populations that are affected by IPV and those who did not experience IPV (Table 1). To facilitate data-driven analysis of conflict in relationships, we used Conflict Tactics Scale (CTS) to cluster a diverse set of participants from studies with different research questions. The participants included in our study are not restricted to victims/survivors or perpetrators of IPV. We included all datasets for which CTS was used and all participants for whom a sufficient number of answers to CTS items were available. These datasets included samples from college students (“Young Adults”^10^, healthy community couples (“Community Sample”^13^), and patients who presented moderate to severe cases of relationship conflict (“Clinical Data”^14^).

**Table 1:** Demographics and characteristics of the datasets that are utilized in this study.

Demographics					
Variable	Statistic	Total (n = 640)	Clinical data (n = 411)	Young adults (n = 148)	Community sample (n = 81)
Gender	Female	383 (59.84%)	246 (59.85%)	93 (62.84%)	44 (54.32%)
	Male	257 (40.16%)	165 (40.15%)	55 (37.16%)	37 (45.68%)
Age	Mean (SD)	30.45 (± 9.75)	32.67 (± 9.63)	22.35 (± 4.88)	34.06 (± 8.99)
	Missing	5 (0.78%)	2 (0.49%)	1 (0.68%)	2 (2.47%)
Race	American Indian or Alaskan Native	7 (1.09%)	5 (1.22%)	1 (0.68%)	1 (1.23%)
	Asian	19 (2.97%)	3 (0.73%)	14 (9.46%)	2 (2.47%)
	Black or African American	21 (3.28%)	10 (2.43%)	11 (7.43%)	0 (0.00%)
	Hispanic or Latino	95 (14.84%)	79 (19.22%)	10 (6.76%)	6 (7.41%)
	Other	35 (5.47%)	28 (6.81%)	5 (3.38%)	2 (2.47%)
	White	455 (71.09%)	285 (69.34%)	102 (68.92%)	68 (83.95%)
	Missing	8 (1.25%)	1 (0.24%)	5 (3.38%)	2 (2.47%)

Measured variables					
Clinical data	Violence (CTS) Relationship Functioning (DAS)	Dyadic Adjustment (DAS) Symptomatology (BSI) Symptomatology (OQ)			
Young adults	Violence (CTS) Dyadic Adjustment (DAS) Emotional Abuse (EAQ) Attachment (ECR, RQ) Coping (CISS) Secure Base (SBNR)	Dominance (DS) Sex Roles (SRES) Sexism (ASI) Jealousy (MDJS) Power (POWER) Affect Regulation (ERC)			
Community sample	Violence (CTS) Relationship Functioning (DAS) Dyadic Adjustment (DAS) Emotional Abuse (EAQ) Attachment (ECR) Stress-Affect Regulation (CISS)	Jealousy (MDJS) Personality (BFI) Health (SF) Trauma (TCS) Stress (FDHI) Dyadic Coping (DCI) Marital Burnout (MB)			

All methods in line with relevant guidelines and regulations of the institutional and/or licensing committee at the time of data collection were followed. All the participants informed consented before the initial data collection. University Hospitals Cleveland Medical Center IRB exempted the requirement of ethical approval for the study as the current study was conducted with the de-identified secondary data.

Overall, the datasets contained a pool of 662 participants with 21 different scales assessing conflict in relationships, as well as personal and social factors. 640 participants were selected from the pool based on the completeness of their answers to the Conflict Tactics Scale (CTS^15^). Namely, 21 participants who answered less than 10% (i.e., 7 out of 78) of CTS items and one participant who had zero variance in responses to CTS items were removed. The responses of the remaining 640 participants to CTS were used to identify subgroups who report similar patterns of violence in their relationships. CTS consists of 78 items in which participants are asked how frequently a given (stated) situation has occurred. It has five subscales: Negotiation (12 items), Verbal Abuse (16 items), Physical Assault (22 items), Sexual Coercion (14 items), and Injury (14 items). For each item, participants select the frequency of occurrence: (1) never happened, (2) happened once or twice, (3) 3–6 times, (4) 7–10 times, (5) 11–20 times, and (6) more than 20 times.

The complete list of scales and subscales that are utilized in our study is below. For each subscale, the number of items within that subscale is shown in parenthesis. ASI: Ambivalent Sexism Inventory^16^. Subscales: Benevolent (11), Hostile (11). BFI: The Big Five Inventory^17^. Subscales: Extraversion (8), Agreeableness (9), Conscientiousness (9), Emotional Stability (8), Openness (10). BSI: Brief Symptom Inventory^18^. Subscales: Somatization (7), Obsession Compulsion (6), Interpersonal Sensitivity (4). CISS: Coping Inventory for Stressful Situations^19^. Subscales: Task (16), Avoidance (3), Social (5), Distraction (8), Depression (6), Anxiety (6), Hostility (5). CTS: Conflict Tactic Scale^15^. Subscales: Negotiation (12), Verbal Abuse (16), Physical Assault (22), Sexual Coercion (14), Injury (14). DAS: Dyadic Adjustment Scale^20^. Subscales: Consensus (13), Affectional Expression (4), Dyadic Satisfaction (10), Dyadic Cohesion (5). DCI: Dyadic Coping Inventory^21^. Subscales: Supportive Dyadic Coping by Partner (5), Negative Dyadic Coping by Partner (4), Supportive Dyadic Coping by Oneself (5), Negative Dyadic Coping by Oneself (4). DS: Dominance Scale^22^. Subscales: Authority (13), Restrictiveness (8), Disparagement (8). EAQ: Emotional Abuse Questionnaire^4^. Subscales: Isolation (24), Degradation (28), Sexual Abuse (7), Property Damage (8). ECR: Experiences in Close Relationships^23^ Subscales: Anxiety (18), Avoid (18). ERC: Emotional Regulation Checklist^24^. Subscales: Lability Negativity (13), Emotional Regulation (10). FDHI: Family Daily Hassles Inventory^25^. Subscales: Time Energy Involvement (20), Negative Involvement (20), Positive Involvement (20). MB: Marital Burnout^26^. Subscale: Marital Burnout (12). MDJS: Multidimensional Jealousy Scale^27^. Subscales: Emotional Jealousy (8), Behavioral Jealousy (8), Cognitive Jealousy (8). Power^28^. Subscales: Partner Power (6), Self-Power (5). RDAS: Revised Dyadic Adjustment Scale (14)^29^. RQ: Relationship Questionnaire^30^ Subscale: Relationship Questionnaire (5). SBNR: Secure Based Narrative Representational^31^. Subscales: Secure base (4). SF: Short Form Health Survey^32^. Subscales: General Health (5), Mental Health (5). SRES: Sex Role Egalitarianism Scale^33^. Subscale: Egalitarian (25). TCS: Trauma Symptom Checklist^34^. Dissociation (6), Anxiety (9), Depression (9), Sati (6), Sleep Disorder (6), Sexual Problems (8).

### Data analysis

#### Assessing pairwise dissimilarity

We created a 640 × 78 data matrix, in which rows represented participants, columns represented CTS items, and each entry represented the respective participant’s response to the respective CTS item. Since we integrated various datasets using different measures, many of the participants had several unmeasured items. In total, 38.8% of the entries in the data matrix were missing. To reliably assess the dissimilarity between all pairs of participants at the presence of a large amount of missing data, we utilized all available items that are common between each pair of participants while computing their dissimilarity. To ensure that the dissimilarity between two participants will not be biased by the number of common items that are available, we used a “NaN-Euclidean”-distance measure. Namely, for a given pair of participants $x$ and $y$, we computed the dissimilarity $d(x,y)$ as follows:

1 $$ d(x,y) = \sqrt {\frac{{\sum\nolimits_{{i \in V_{x,y} }} {(x_{i} - y_{i} )}^{2} }}{{|V_{x,y} |}}} $$

where $V_{x}$ denotes the set of items that are non-missing for participant $x$, $V_{y}$ denotes the set of items that are non-missing for participant $y$, and $V_{x,y} = V_{x} \cap V_{y}$ denotes the set of items with non-missing values for both $x$ and $y$. The results of a simulation study that verifies the unbiased nature of this NaN-Euclidian measure of dissimilarity is provided in Supplementary Fig. 2.

#### Hierarchical clustering

Using the pairwise dissimilarity matrix computed based on the dissimilarity measure in Eq. (1), we performed agglomerative hierarchical clustering of the participants. We used the *linkage* function in MATLAB by specifying Ward’s minimum variance method as the criterion for merging clusters^35^. This process led to a hierarchical tree of clusters, in which the root represents all participants, and each leaf represents an individual participant.

#### Selection of subgroups

An important challenge in interpreting the results of hierarchical clustering is selecting the “subgroups” that are potentially representative of IPV subtypes. In selecting the final clusters, an important criterion is the homogeneity of a cluster, i.e., how representative a cluster is in terms of the similarity of the participants in the cluster to each other. To assess the homogeneity of a cluster *C*, we used the *mean pairwise distance* between all participants in the cluster, i.e.,

2 $$ \overline{d}(C) = \sum\limits_{x,y \in CxC} {\frac{d(x,y)}{{\left( {\frac{|C|}{2}} \right)}}} $$

where $|C|$ denotes the number of participants in cluster $C$. Lower (higher) $\overline{d}(C)$ values correspond to a more (less) similar group of participants in $C$ based on their responses in CTS, indicating higher cluster homogeneity (heterogeneity).

**Figure 2:**
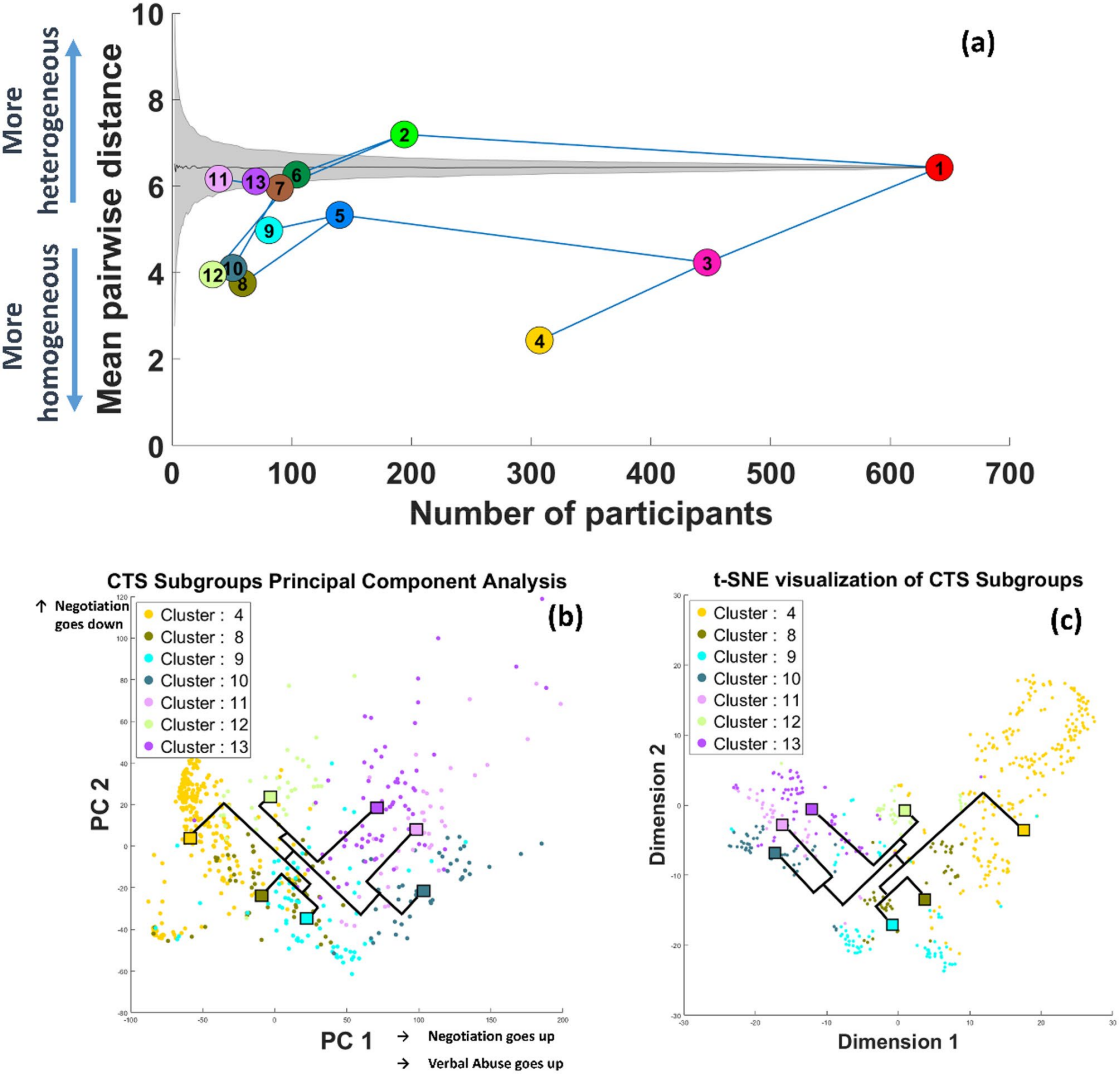
Identification of intimate partner violence subgroups based on hierarchical cluster analysis using integrated data on Conflict Tactics Scale. Data from 640 participants from 5 different studies were integrated to hierarchically cluster the participants. (**a**) Visualization of the clustering in the space of cluster size (number of participants in each cluster) vs. cluster heterogeneity (mean pairwise distance). The tree structure shows the structure of the relationship between the clusters in the context of hierarchical clustering, where the red node represents the root cluster, i.e., all participants. The leaf nodes of the tree show the distinct subgroups, i.e. the clusters that are identified as potentially representative of subtypes. (**b**) The relationship between the clusters that are representative of subtypes in the two-dimensional space represented by the first two principal components (PCA) of the data matrix. (**c**) The relationship between the clusters that are representative of subtypes in the two-dimensional representation computed using t-stochastic neighbor embedding (t-SNE). In these figures, each dot shows a participant, the color of the dot represents the assigned cluster for the participant, and the diamonds represent the cluster centroids. For the PCA visualization, axes are labeled by the interpretation of the respective principal components based on the loadings of the subscales on each principal component.

We visualized the hierarchical clustering in the space of cluster size vs. cluster heterogeneity (mean pairwise distance, Fig. 2a). Then, to obtain a baseline for cluster heterogeneity, we performed permutation tests by constructing subsets of randomly selected participants and computing the mean pairwise distance in these random subsets. Repeating this procedure for each cluster size, we estimated the expected mean and standard deviation of the cluster heterogeneity for random clusters with a given size (seen as a gray cloud in Fig. 2a). This allowed us to provide a context for the evaluation of the homogeneity of the clusters identified via hierarchical clustering. Finally, to determine the final set of clusters that represent subgroups, we applied the following principle:

1. if a cluster is sufficiently large (containing more than 50% of all participants), always allow a split i.e., always divide the cluster into two child clusters (Fig. 1d, right panel).
2. If a cluster is sufficiently small (containing less than 10% of all participants), never allow a split i.e., keep the cluster as a subgroup (Fig. 1d, left panel).
3. Otherwise, allow a split (divide into child clusters) if the split benefits cluster homogeneity, i.e. if both children are more homogeneous than their parent cluster (Fig. 1d, middle panel).

#### Identification of subgroup characteristics

After the set of subgroups were finalized, we annotated them using CTS in the context of the hierarchy of clustering. For this purpose, we identified significant items that play an important role in the split of clusters. For each item $i$, we compared the mean values of item $i$ in each pair of sibling clusters $C_{j}$ and $C_{k}$ using two-sample student’s t-test:

3 $$ t_{i} = \frac{{u_{i} (C_{j} ) - u_{i} (C_{k} )}}{{\sqrt {\frac{{\sigma_{i} (C_{j} )^{2} }}{{n_{j} }} + \frac{{\sigma_{i} (C_{k} )^{2} }}{{n_{k} }}} }} $$

where $u_{i} (C_{j} )$ and $\sigma_{i} (C_{j} )$ respectively denote the mean and the standard deviation of item $i$ in cluster $C_{j}$ and $n_{j}$ denotes the number of participants in cluster $C_{j}$.

We employed the statistical framework for Gene Set Enrichment Analysis (GSEA), which aims to identify sets of items that *collectively* exhibit differentiation between two groups of samples^36^. Enrichment analysis was used to identify subscales (sets of items) that are enriched in items with high differentiation between sibling clusters. For each cluster split {$C_{j}$, $C_{k}$} and each subscale $S$, we used enrichment analysis to assess whether items in $S$ tend to have significantly large t-statistics (positive or negative) for the split, in comparison to all other items in CTS.

**Figure 3:**
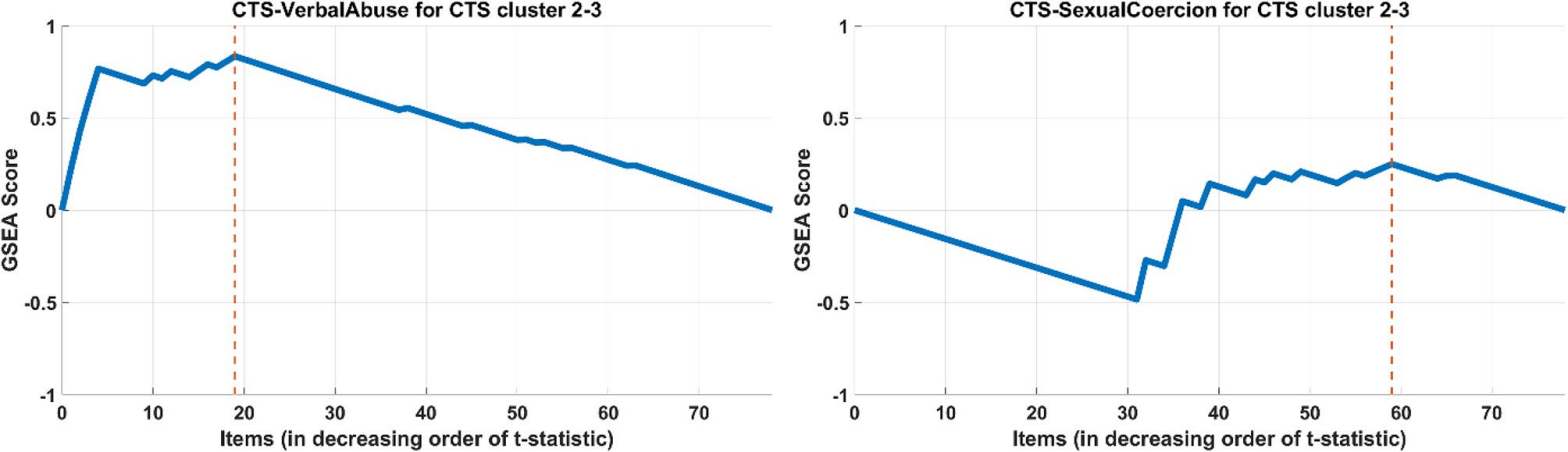
Illustration of enrichment analysis on the split of Clusters “2” and “3” for Verbal Abuse and Sexual Coercion subscales. The y-axis shows the enrichment score for the respective subscale as a function of the ranking of items in CTS in terms of their differentiation between clusters “2” and “3”. Verbal Abuse has a higher enrichment score than Sexual Coercion since items in the Verbal Abuse subscale are ranked higher according to their differentiation with respect to this split.

To compute the enrichment score of $S$ with respect to split $C_{j}$ vs. $C_{k}$, we first ranked all items in CTS according to the absolute values of their t-statistics, in descending order, i.e. we sorted the items in such a way that if $a < b$, then we have $\left| {t_{a} (C_{j} ,C_{k} )} \right| \le \left| {t_{b} (C_{j} ,C_{k} )} \right|$. Then, we calculated the enrichment score of $S$ as a running sum over this ranked list of t-statistics, which increases when we encounter an item in subscale $S$ (called a *hit*), and decreases when we encounter an item not in subscale $S$ (called a *miss*). The enrichment score for subscale $S$, denoted $ES(S)$, was then computed as the peak value of this running sum, i.e.:

4 $$ ES_{hit} (S,i) = \sum\limits_{a \in S,a \le i} {\frac{{|t_{a} |}}{T}} ,\quad where \, T = \sum\limits_{a \in S} {|t_{a} |} $$

5 $$ ES_{miss} (S,i) = \sum\limits_{a \le i} {\frac{1}{N - |S|}} $$

6 $$ ES(S) = \mathop {max}\limits_{i} (ES_{hit} (S,i) - ES_{miss} (S,i)) $$

here, $N$ denotes the total number of items that are considered (all items in CTS). This process is visualized for two different subscales of CTS with respect to the first split in the hierarchical clustering of the participants (Fig. 3).

We assessed the significance of these enrichment scores by computing p-values using permutation tests. For each cluster split (into two subgroups) and a subscale, we aimed to test the following null hypothesis with these permutation tests: “Given the differences of the means of all items in CTS between the two subgroups (as quantified using t-statistics), the enrichment score of the set of items in the subscale is not larger than what can be expected for a random set of items with size equal to that of the subscale.” In other words, the null hypothesis states that the subscale is not enriched in items that are reported at different levels by the participants in the two subgroups. To test this hypothesis, we generated 1000 permutations by selecting $S$ items randomly from the set of all items in CTS. For each of these random item sets (denoted $S^{(l)}$ for $1 \le l \le 1000$), we computed the enrichment score $ES(S^{(l)} )$. Then, we computed the empirical p-value $p(S)$ as the fraction of permutations with a higher enrichment score than the observed $ES(S)$ i.e.:

7 $$ p(S) = \frac{{|\{ 1 \le l \le 1000:ES(S^{(l)} ) \ge ES(S)\} |}}{1000} $$

Starting from the root of the dendrogram, we applied enrichment analysis to each split in the clustering hierarchy to identify CTS subscales that are significantly enriched in highly differentiated items between the splits. We reported the significance of the enrichment of the subscales at three levels of significance threshold: individual test level (p < 0.05), intermediate level (p < 0.01), and stringent level that accounts for multiple hypothesis testing (p < 0.001). The stringent threshold corresponds to the Bonferroni-corrected version of the threshold for individual tests, as the number of subscales tested at each level was less than or equal to 50. The number of subscales tested went down as we went down in the hierarchy, since the number of participants were smaller in the corresponding subgroups, thus sufficient data was not available for some subscales.

#### Projection to other scales

We investigated the main differences between subgroups using items measured by multiple questionnaires. These scales measure various factors including personal and relationship factors and mental health. We also used enrichment analysis to test the enrichment of each non-CTS subscale against all non-CTS subscales with respect to each split in the dendrogram. For these subscales, we tested the following null hypothesis for each cluster split in the hierarchy (into two subgroups): “Given the differences of the means of all items in all scales other than CTS between the two subgroups (as quantified using t-statistics), the enrichment score of the set of items in the subscale is not larger than what can be expected for a random set of items with size equal to that of the subscale.” Thus, a smaller p-value indicates more significant enrichment of the non-CTS subscale in items that are reported at different levels by the participants in the two subgroups. Since these subscales were not used in the identification of subgroups, the significant subscales we identified in this analysis served as an additional validation for the subgroups and provided further information for the annotation of the subgroups.

#### Effect size for subscales

As discussed above, we use enrichment analysis to identify subscales that exhibit differential reporting between two subgroups, for both CTS and non-CTS subscales. Since enrichment analysis is rather complex, the enrichment score is not interpretable as effect size. For this reason, to facilitate interpretable analysis with easy to understand effect size estimations of the differences between the groups, we used two additional measures. Specifically, for each pair of groups in the hierarchical tree and for each subscale, we applied one of the following two procedures depending on the rarity of the subscale: (1) *Prevalence:* For uncommon subscales with infrequently reported items, we assigned each participant to a binary value (i.e., reported/not reported, we assigned the binary value as 1 if any of the items in that subscale have a non-zero response regardless of their quantitative values, and 0 otherwise). Then we computed the average of these for the participants in the groups and reported as the prevalence for each subgroup. (2) *Reported Average:* For relatively common subscales, we measured the mean value (across all items in the subscale and participants in the group) and provided them as “reported average” for each subgroup.

## Results

### Hierarchical clustering of participants

The results of agglomerative hierarchical clustering analysis are shown in Fig. 2. In Fig. 2a the horizontal location of a cluster shows the clusters’ size and the vertical location shows its mean pairwise distance. Cluster “1” in the figure corresponds to all participants, while the clusters “2” and “3” represent its “children” (hence the union of clusters “2” and “3” is the set of all participants). As can be seen in the figure, cluster “3” is more homogeneous than it would be expected at random, whereas cluster “2” is more heterogeneous than random groups of participants of the same size as itself. Using this visualization and the criteria described above we selected clusters “4”, “8”, “9”, “11”, “12” and “13” as “leaf” clusters that represent subgroups. These subgroups (clusters) respectively contain 306, 59, 81, 51, 39, 34, and 70 participants, with cluster “4” being the most homogeneous and clusters “11” and “13” being the most heterogeneous.

### Visualization of clusters in reduced dimensional space

To visually assess the coherence of the resulting subgroups, we visualized the participants in all subgroups in reduced dimensional space obtained using Principal Component Analysis (PCA) and t-Distributed Stochastic Neighbor Embedding (t-SNE). In these figures, the participants and cluster centroids are shown as points in the 2-dimensional space defined by the two most significant principal components of the data matrix (Fig. 2b) and t-SNE’s optimization function that aims to preserve local patterns in data (by setting the perplexity parameter to 30, Fig. 2c). As seen in the figure, participants that belong to the same subgroup are typically closer in the two-dimensional space computed by both PCA and t-SNE. We also observe that the position of cluster centroids in the reduced dimensional PCA and t-SNE spaces are consistent with the hierarchical organization of the clusters, and with each other. The top two principal components were interpreted based on their loadings in the subscales; the “Negotiation” subscale dominated both dimensions, whereas “verbal abuse” contributed to the most dominant principal component.

### Interpretation of cluster splits

**Figure 4:**
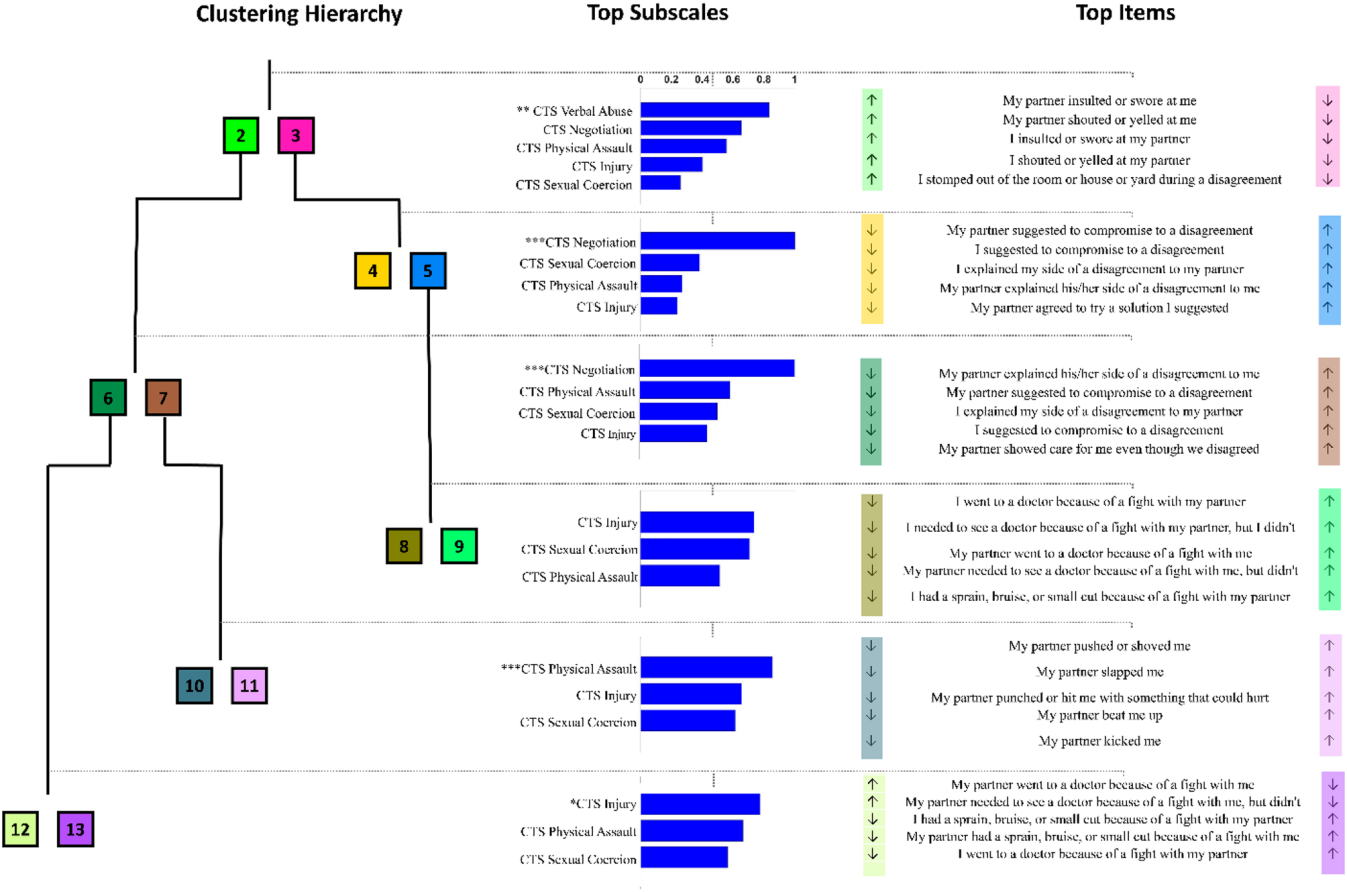
The items and subscales of Conflict Tactics Scale (CTS) that drive the difference between each cluster split in the hierarchical representation of intimate partner violence (IPV) subgroups. The left panel shows the subgroups identified using hierarchical clustering on CTS within the context of the hierarchical clustering. Each box represents a subgroup with a color assigned to it. For each split in the hierarchy, the bars in the middle panel that are horizontally aligned with the split show enrichment scores of the top CTS subscales separating the corresponding sibling clusters. The statistical significance of these enrichment scores based on permutation tests are indicated with stars: ***p < .001, **p < .01, *p < .05. The right panel lists the five features with the highest absolute t-statistic from the most significant subscale for each cluster split. The arrows that are next to the items indicate which cluster (left or right) have higher or lower values for the corresponding question. For example, the arrows next to the question “*My partner insulted or swore at me*” indicate that participants in Cluster “2” typically reported this item more frequently than the participants in Cluster “3”.

The subscales of CTS that are significantly different between sibling clusters in the context of hierarchical clustering (as identified using enrichment analysis) are shown in Fig. 4. To focus on subscales that are specific to each split (rather than those inherited from the parent split), we removed the subscales that have a statistically significant enrichment score ($p < 0.05$) from the analysis of a child clustering split. For example, since the “CTS—Verbal Abuse” subscale is significant for the “2”–“3” cluster split ($p < 0.001$), it is not considered for the enrichment analysis involving the “4”–“5” cluster split.

We observed that the following subscales were most characteristic to each cluster split: Verbal Abuse was most significant for split “2”–“3” (p < 0.001, 10.8 vs. 1.4 reported average), with participants in cluster “2” reporting more verbal abuse. Negotiation was most significant for the split of these two clusters (cluster “2” into “6”–“7” (p < 0.001, 9.2 vs. 20.5 reported average) and cluster “3” into “4”–“5” (p < 0.001, 4.9 vs. 17.0 reported average). Participants in cluster “5” reported more negotiation as compared to those in cluster “4”, while participants in cluster “7” reported more negotiation as compared to those in cluster “6”.

We were also able to identify significant subscales for the differentiation of the children of cluster “7” (with participants in cluster “11” reporting more physical assault from their partners than those in cluster “10”, p < 0.001, 0.64 vs. 2.26 reported average) and that of cluster “6” (with participants in cluster “12” reporting more injury caused by partners than participants in cluster “13” p < 0.05, 0.18 vs. 0.41 reported average). No subscale was significantly enriched in the split of cluster “5” into clusters “8” and “9”, but Injury (p = 0.08, 0.03 vs. 0.10 reported average) and Sexual Coercion (p = 0.14, 0.26 vs. 0.52 reported average) subscales were the highest-ranked subscales for this split, with participants in cluster “9” reporting more injury and sexual coercion.

In the split between clusters “2” and “3”, participants in cluster “2” reported more verbal abuse. Complete enrichment results of CTS for all splits are shown in Supplementary Table 1.

### Projection of clustering on other scales

**Figure 5:**
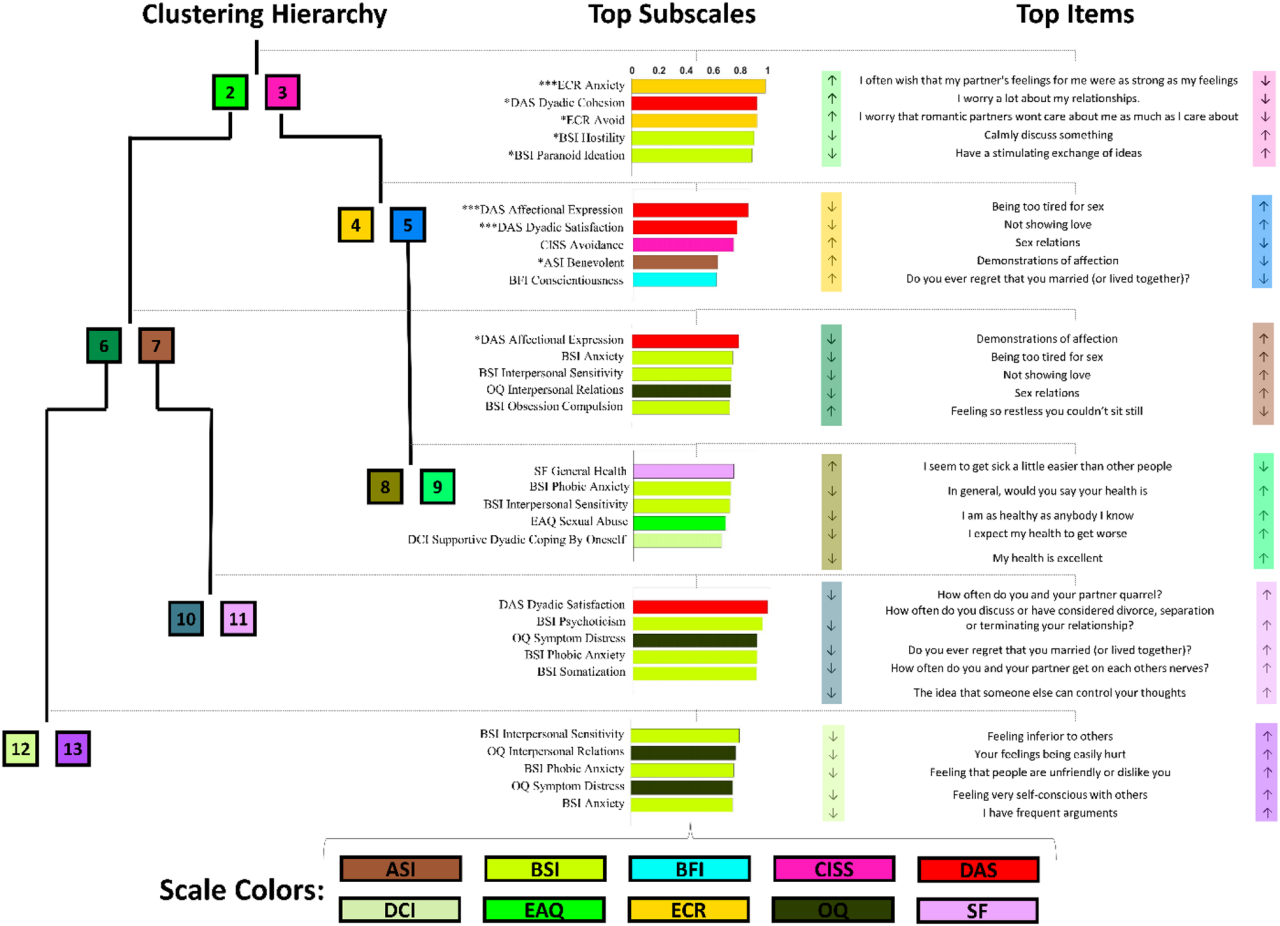
Identification of subscales of non-CTS scales (listed in Table 1) significantly splitting the clusters of CTS. Similar to Fig. 4, the left panel shows the hierarchical structure of the identified clusters (based on CTS questions). The middle panel lists the top five subscales separating cluster splits among remaining 72 subscales in terms of their enrichment scores. The right panel lists the top one questionnaire item for each subscale listed in the middle panel. Note that, in the middle panel, the color indicates the scale of the corresponding subscale.

To understand whether the subgroups identified using CTS also had other distinguishing characteristics, we investigated the differentiation of subscales of other scales. For this purpose, we used enrichment analysis as we did for the CTS subscales for the annotation of subgroups. The results of this analysis are shown in Fig. 5. We observed that a large number of subscales were significantly differentiated between clusters “2” and “3”, where participants in cluster “2” reported more anxiety (ECR, p < 0.001, 84% vs 71% prevalence), more avoidance (ECR, p < 0.01, 72.3% vs 61.5% prevalence), less dyadic cohesion (DAS, p < 0.05, 2.71 vs. 3.08 reported average), less consensus (DAS, p < 0.05, 3.16 vs. 3.75 reported average), more hostility (BSI, p < 0.05, 40.9% vs. 24.2%, more paranoid ideation (BSI, p < 0.05, 34.4% vs. 20.8% prevalence), and more depression (BSI, p < 0.05, 39.6% vs. 27.3% prevalence), as compared to participants in cluster “3”. Multiple subscales were also enriched in the split of cluster “3”, where participants in cluster “4” reported more affectional expression (DAS, p < 0.001, 3.83 vs. 3.40 reported average), more dyadic satisfaction (DAS, p < 0.001, 2.81 vs. 1.72 reported average), and more benevolent sexism (ASI, p < 0.05, 2.91 vs. 2.38 reported average) as compared to participants in clusters 5. The other split for which affectional expression (DAS) was significant was the split of cluster “2” into clusters “6” and “7”, where participants in cluster “6” reported more affectional expression (DAS, p < 0.001, 2.66 vs. 3.17 reported average). Complete enrichment results of all the other subscales for all splits are shown in Supplementary Table 2.

## Discussion

The main objective of this study was to identify relatively homogeneous subgroups of individuals with respect to the forms and severity of violence perpetrated in their relationship. The multi-dimensional perpetration forms we considered included verbal, physical, injurious, and sexual components of violence. Our findings indicated that, in general, subgroups of cases with more violence (in terms of severity and the types of violence that are presented) tend to be more heterogeneous than subgroups of cases with less violence.

We found that emotional abuse was the first distinctive feature among subgroups, followed by sexual coercion. This finding is consistent with past research indicating that emotional abuse and sexual coercion have differential implications on subtyping of IPV^8,37^. As previously reported in the literature, the distinctive feature among the most severe cases was injury^9^. Overall, our study represents an important step toward using integrative data analysis to understand the etiology and the complexity of IPV.

The main division of all cases (both non-violent relationships and violent relationships) into two subgroups was primarily based on the presence of verbal abuse. The difference between these two subgroups also manifested in other personal and relationship characteristics. In particular, we found that the subgroup with more verbal abuse consisted of individuals with more anxiety, avoidance, hostility, and paranoid ideation while reporting less dyadic cohesion.

Both of the two main subgroups were split along the lines of the presence of negotiation. Interestingly, however, the presence of negotiation seemed to have different interpretations in each subgroup. Namely, the subgroup with more verbal abuse was divided into two subgroups, where the subgroup with less negotiation was associated with more physical violence. In contrast, the subgroup with less verbal abuse was divided into two subgroups, such that the subgroup with more negotiation was associated with more relationship conflict. We call this observation “reversal of negotiation”, in that the presence of negotiation is associated with the presence of conflict in a relationship. However, if the conflict is present, absence of negotiation is associated with the presence of violence. This observation suggests that some forms of violence can be prevented by promoting negotiation in high-conflict relationships. There exist group couple therapy models that focus on skill building for communication and negotiation^38,39^. Further investigation of this pattern can provide more insights into the efficacy of these therapy models and enable identification of relationships for which such treatments can be effective.

We also observed that as we went down the hierarchy of clustering, (1) the scale of the severity of violence in distinguishing clusters became larger, and (2) the types of violence that distinguished the clusters became more variable. In total, our data-driven approach indicated twelve hierarchically organized subtypes of IPV.

### Two main subgroups split by verbal abuse

When we focused on the separation of the set of all participants into two subgroups, the branching was based on their frequency of verbal abuse during arguments. These included degradation, name-calling or other behaviors that target the psychological well-being of the partner. The subgroup that reported less frequent verbal abuse (cluster “3”, 446 participants) was more homogeneous than the subgroup with more frequent verbal abuse (cluster “2”, 194 participants), despite being larger.

In the next level of branching, participants in clusters “2” and “3” were both split into two subgroups based on their efforts in negotiation. Cluster “3” was split into a more homogeneous subgroup with lower levels of negotiation (cluster “4”, 306 participants) and a smaller, but more heterogeneous subgroup with higher levels of negotiation (cluster “5”, 140 participants). In general, the participants in cluster “5” reported higher levels of conflict in their relationship than those in cluster “4”. The split of cluster “2” followed a pattern parallel to that of cluster “3” (Fig. 2b), but both subgroups that branched from cluster “2” were more homogeneous than cluster “2” itself. In contrast to the split of cluster “3”, the subgroup of cluster “2” that reported lower levels of negotiation (cluster “6”, 104 participants) reported higher levels of relationship conflict than the subgroup that reported higher levels of negotiation (cluster “7”, 90 participants). As discussed above, this “reversal of negotiation” indicates the level of conflict at which negotiation is not effective in reducing relationship conflict.

When we investigated the characteristics of subgroups in terms of their projection to other scales, our results indicated that the main division was between these two main subgroups. This analysis indicated that cluster “3”, which is relatively homogeneous with respect to CTS, is also homogeneous with respect to other scales in that participants are highly similar to each other. Participants in this subgroup reported high cohesion and consensus in their dyadic adjustment. In contrast, participants in cluster “2” reported less relationship cohesion and consensus, while they also reported higher levels of anxiety, avoidance, depression, hostility, and paranoid ideation. Overall, people in cluster “2” appear to have more mental health issues and lower attachment security. This is in agreement with previous literature reporting that mental health issues such as depression and anxiety, as well as attachment security, are major risk factors for IPV ^8,10,11^.

### Subgroups with less verbal abuse

Subgroups “4” and “5” were branching out from subgroup “3”. Even though there was no severe violence in these subgroups, there were significant differences in relationship atmosphere. Participants in subgroup “4” reported higher levels of affectional expression of love and caring, positive involvement when faced with hardship, and higher dyadic satisfaction. Interestingly, for subgroup 4, benevolent sexism was also higher and distinctive. This might be because benevolence has a moderating role with relationship satisfaction and hurtful partners as long as they are not challenged with relationship problems and hurtful partner behaviors^35^. However, more research is needed to understand the role of benevolent sexism in IPV.

Subgroup “5” branched out into further subgroups “8” and “9”. Although it did not reach a statistically significant level, subgroup “9” showed a tendency for more task-oriented coping and having a positive involvement with stressful situations. This might be linked with less relationship conflict, along with tendencies to avoid arguments and cope by focusing on solutions. This subgroup has a resemblance to Gottman’s subtype of conflict-avoiding couples, those characterized by minimal emotional expressiveness, and having clear boundaries^40,41^.

### Subgroups with more verbal abuse

Subgroup “2” branched out into subgroups “6” and “7”. We observed that cluster “6” was composed of participants who reported the most severe violence in their relationship, where the two subgroups that branched from cluster “6” were mainly separated by the presence of injury. The subgroups that branched from cluster “7”, on the other hand, were mainly separated by the presence of physical assault. These subtypes of violence seem to be both quantitatively and qualitatively different from each other in terms of the atmosphere they created in the relationship.

Those in subgroup “6” (lower levels of negotiation) reported higher levels of interpersonal problems and difficulty in forming relationships. They were more likely to report feeling lonely, as well as unloved, and unwanted by their partners and family members. Interestingly, however, participants in subgroup “7” reported having less affectionate expression for their partners as compared to those in subgroup “6”.

Subgroups “10” and “11” branched further out from subgroup “7”. Our results indicated that participants in subgroup “10” form a relatively homogeneous cluster. Participants in this subgroup reported the highest level of physical assault. As seen in Fig. 2b, the level of verbal abuse was also higher for subgroup “10” compared to subgroup “11”. Behaviors like name-calling, insulting, and other forms of verbal abuse were more commonly and severely observed. High conflict in this subgroup is also paired with higher effort for negotiation. Subgroup “11”, on the other hand, was relatively more heterogeneous. While this subgroup also reported high levels of conflict, verbal abuse and efforts for negotiation were lower. Although there were disagreements, participants in this subgroup did not necessarily engage in verbal aggression or negotiation. Projection on other scales indicated that those in subgroup “11” carried more regret in their relationship, discussed the termination of relationship, and complained about their partners. These results are also in line with literature indicating that individuals who suffer from IPV and are not good with repairing the relationship are more likely to experience relationship instability, and dissolving relationships are far more common than in nonviolent relationships^42,43^.

The final groups branching out from subgroup “6” were subgroups “12” and “13”. Subgroup “12”, the smallest group in our study, reported the highest levels of injury, with visits to emergency departments and medical facilities due to violent altercations. This subgroup also experienced lower anxiety and interpersonal sensitivity compared to subgroup “13”. In subgroup “12” participants were older and had lower education levels. There were also more women (76%) in this subgroup (Supplementary Fig. 1).

### Implications for IPV treatment

We observed different subtypes of violence in regard to frequency, severity, and forms. Emotional abuse is the main source of distinction, followed by sexual coercion, and the most severe cases are associated with injury. The results of this study could be utilized to channel patients into treatments based on their needs. Usually. the treatments are offered under the umbrella concept of intimate partner violence. The majority of IPV treatments emphasize heavily on physical aspects of violence with interventions focusing on cognitive behavioral interventions as well as safety planning and time-out techniques^44^. These interventions are critical for the safety of the victims particularly for severe partner violence (Subgroup 13). However, for subgroups 10, 11, and 12, treatments specifically focusing on emotional abuse and underlying needs such as anxiety and stress might also be helpful. Furthermore, there are limited number of treatments available specifically for emotional and sexual abuse/violence in the intimate relationships. The development of more targeted treatment options specific to these subgroups might improve the treatment efficacy.

### Limitations and future work

Distinguishing subtypes of domestic violence instances is critical to developing a deeper understanding of not only the causal factors for violence, but also for guiding the treatment efforts^38^. While the subgroups we identify in this study can be useful in guiding treatment efforts, the datasets included in our analyses did not include treatment information. Replication of this type of analysis on richer datasets with treatment information can therefore provide more insights into the relationship between subgroup characteristics and efficacy of treatment models.

The datasets we analyzed in this study included data from victims/survivors, perpetrators, and those who did not report significant conflict or violence in their relationship. This approach was motived by our objective of providing an unbiased data-driven approach to identifying subgroups of intimate partner violence. To this end, our results should be interpreted considering the unsupervised nature of our analyses. Development of frameworks that aim to identify subgroups of perpetrators, victims/survivors, and relationships within dyadic contexts can potentially provide additional insights into subtypes of IPV.

Finally, the datasets we integrated in this study did not include same-sex couples. Generalization of these integrative data analysis frameworks to same-sex couples will also be essential in understanding the etiology of IPV in same-sex couples and development of treatment models specifically targeting same sex couples.

In conclusion, the subgroups we identified with this integrative approach provide new insights into the manifestation of violence in different relationships and can provide assistance in the development and selection of treatment programs. The methodology we developed is also generalizable to other problems in psychology and behavioral sciences that will benefit from integrative data analyses.

## Supplementary Information

Supplementary Information.
