# Supplementary material for: Data-driven identification of subtypes of intimate partner violence
Source: Sci Rep. 2021 Mar 24;11:6736. doi: 10.1038/s41598-021-85947-3 (PMC7991636; doi:10.1038/s41598-021-85947-3)
Supplement: Supplementary file 1 — Supplementary Information. [file 41598_2021_85947_MOESM1_ESM.docx]

**Supplement to**

**“Data-driven identification of subtypes of intimate partner violence”**

Ahmet Hacıaliefendioğlu, Serhan Yılmaz, Douglas Smith,

Jason Whiting, Mehmet Koyutürk, and Günnur Karakurt





**Supplementary Figure 1. Demographics of the identified subgroups of CTS.** Rows indicate different demographic characteristics, namely age, education level, race, and gender. Columns indicate different subgroups which are identified based on CTS responses. The left-most column represents all 640 participants. The numbers in each colored block indicate the number of participants in the subgroup having the specified demographic characteristic.


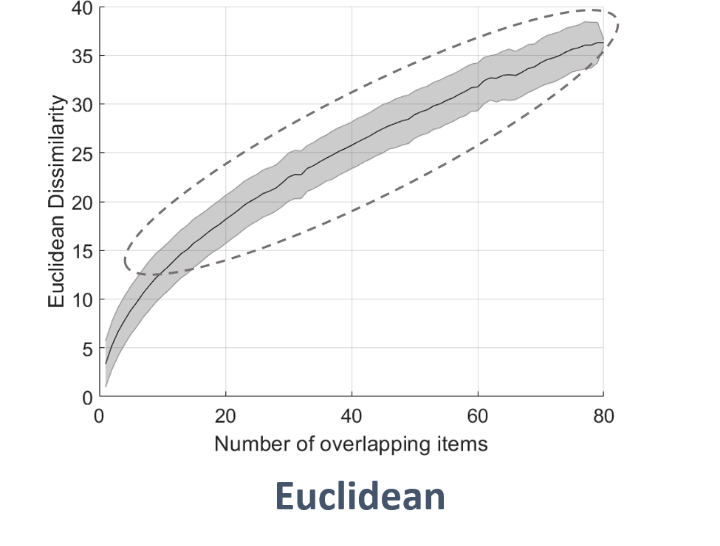

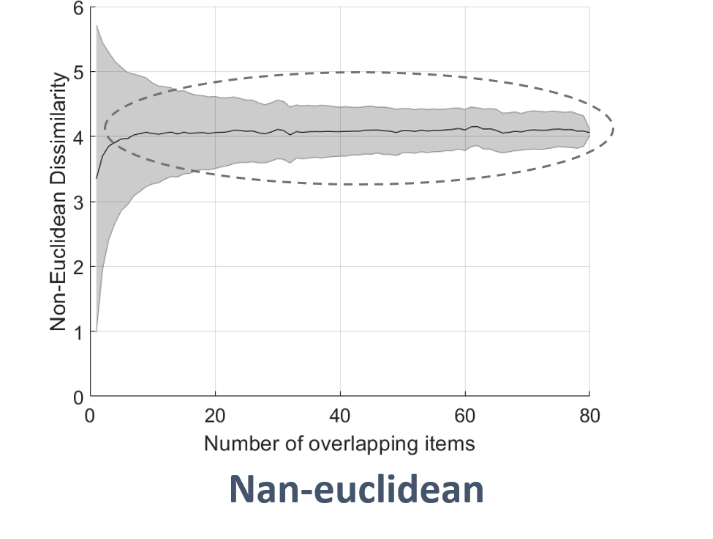
**Supplementary Figure 2. NaN-Euclidian measure of dissimilarity removes bias caused by varying number of common dimensions between samples.** In our simulation study, we generated a 640x78 data matrix in which rows represent samples (participants) and columns represent dimensions (items). The entries in the matrix were taken from a uniform distribution. Then, we randomly selected and removed 38.8% of the entries in the matrix, to create missing (NaN) data in the same amount as in the original data.  We then computed the dissimilarity between all pairs of using Euclidean distance (without normalization) and NaN-Euclidean distance that we use to take into account missing values and the number items that are used to assess the distance between each pair of samples (Equation 1). In each panel, the x-axis shows the number of common (overlapping) dimensions between the pair of samples and the y-axis shows the dissimilarity computed using Euclidian distance (left) and NaN-Euclidian distance (right). The black lines show the mean value across all sample pairs with the respective number of overlapping dimensions, and the shaded areas indicate the range for two standard deviations. As it can be seen, unlike the Euclidean distance, we do not observe considerable bias or variance in NaN-Euclidian dissimilarity with varying number of overlapping items between samples. The mean dissimilarity is lower and there is high variance when the number of overlapping dimensions is very small (e.g., smaller than 5), which is not common in our study since the participants included in the analysis have answers for at least 7 of the 78 items.

**Supplementary Table 1. Enrichment analysis on CTS subscales for the annotation of subgroups.** The enrichment scores (abbreviated E.S.) and significance figures (p-values) for each CTS subscale are listed for each split in the context of hierarchical clustering.

| Scale | Subscale | Split 2-3 | | Split 4-5 | | Split 6-7 | | Split 8-9 | | Split 10-11 | | Split 12-13 | |
| --- | --- | --- | --- | --- | --- | --- | --- | --- | --- | --- | --- | --- | --- |
|  |  | E.S. | P-value | E.S. | P-value | E.S. | P-value | E.S. | P-value | E.S. | P-value | E.S. | P-value |
| CTS | Injury | 0.403 | .965 | 0.337 | .984 | 0.436 | .993 | 0.734 | .081 | 0.712 | .587 | 0.771 | .026* |
| CTS | Negotiation | 0.654 | .408 | 1 | .001*** | 1 | .001*** | - | - | - | - | - | - |
| CTS | Physical Assault | 0.575 | .666 | 0.313 | 1 | 0.583 | .941 | 0.511 | .811 | 0.929 | .001*** | 0.662 | .208 |
| CTS | Sexual Coercion | 0.249 | .997 | 0.355 | .97 | 0.5 | .959 | 0.704 | .145 | 0.667 | .781 | 0.562 | .63 |
| CTS | Verbal Abuse | 0.834 | .005** | - | - | - | - | - | - | - | - | - | - |

**Supplementary Table 2. Enrichment analysis on the subscales of non-CTS scales for the projection of CTS subgroups to other factors.** The enrichment scores (abbreviated E.S.) and significance figures (p-values) for each subscale of non-CTS scales are listed for each split in the context of hierarchical clustering.

| Scale | Subscale | Split 2-3 | | Split 4-5 | | Split 6-7 | | Split 8-9 | | Split 10-11 | | Split 12-13 | |
| --- | --- | --- | --- | --- | --- | --- | --- | --- | --- | --- | --- | --- | --- |
|  |  | E.S. | P-  value | E.S. | P-  value | E.S. | P-value | E.S. | P-value | E.S. | P-value | E.S. | P-value |
| ASI | Benevolent | - | - | 0.724 | .028* | - | - | - | - | - | - | - | - |
| ASI | Hostile | - | - | 0.633 | .125 | - | - | - | - | - | - | - | - |
| BFI | Agreeableness | 0.502 | .836 | 0.696 | .072 | - | - | 0.471 | .936 | - | - | - | - |
| BFI | Conscientiousness | 0.493 | .853 | 0.716 | .064 | - | - | 0.641 | .371 | - | - | - | - |
| BFI | Emotional Stability | 0.509 | .829 | 0.651 | .169 | - | - | 0.598 | .543 | - | - | - | - |
| BFI | Extraversion | 0.428 | .962 | 0.687 | .107 | - | - | 0.623 | .438 | - | - | - | - |
| BFI | Openness | 0.609 | .498 | 0.253 | .984 | - | - | 0.661 | .255 | - | - | - | - |
| BSI | Anxiety | 0.69 | .328 | 0.51 | .521 | 0.929 | .234 | 0.524 | .809 | 0.885 | .469 | 0.894 | .394 |
| BSI | Depression | 0.898 | .019* | - | - | - | - | - | - | - | - | - | - |
| BSI | Hostility | 0.919 | .022* | - | - | - | - | - | - | - | - | - | - |
| BSI | Interpersonal Sensitivity | 0.71 | .359 | 0.502 | .57 | 0.918 | .223 | 0.787 | .187 | 0.87 | .42 | 0.953 | .13 |
| BSI | Obsession Compulsion | 0.704 | .325 | 0.346 | .875 | 0.9 | .361 | 0.51 | .829 | 0.896 | .426 | 0.893 | .406 |
| BSI | Paranoid Ideation | 0.904 | .034* | - | - | - | - | - | - | - | - | - | - |
| BSI | Phobic Anxiety | 0.673 | .393 | 0.241 | .964 | 0.874 | .446 | 0.792 | .125 | 0.904 | .384 | 0.904 | .326 |
| BSI | Psychoticism | 0.755 | .247 | 0.485 | .612 | 0.879 | .419 | 0.593 | .632 | 0.943 | .184 | 0.869 | .487 |
| BSI | Somatization | 0.649 | .448 | 0.291 | .929 | 0.869 | .578 | 0.546 | .768 | 0.9 | .459 | 0.865 | .626 |
| CISS | Avoidance | 0.426 | .876 | 0.855 | .059 | - | - | 0.479 | .827 | - | - | - | - |
| CISS | Distraction | 0.44 | .937 | 0.578 | .303 | - | - | 0.494 | .884 | - | - | - | - |
| CISS | Emotion | 0.466 | .93 | 0.375 | .799 | - | - | 0.515 | .791 | - | - | - | - |
| CISS | Social | 0.532 | .766 | 0.573 | .392 | - | - | 0.553 | .719 | - | - | - | - |
| CISS | Task | 0.548 | .654 | 0.421 | .652 | - | - | 0.656 | .145 | - | - | - | - |
| DAS | Affectional Expression | 0.867 | .087 | 0.989 | .001*** | 0.986 | .04* | - | - | - | - | - | - |
| DAS | Consensus | 0.774 | .033* | - | - | - | - | - | - | - | - | - | - |
| DAS | Dyadic Cohesion | 0.935 | .015* | - | - | - | - | - | - | - | - | - | - |
| DAS | Dyadic Satisfaction | 0.683 | .274 | 0.895 | .001*** | 0.889 | .514 | - | - | 0.98 | .07 | - | - |
| DCI | Negative Dyadic Coping By Oneself | 0.859 | .091 | 0.223 | .971 | - | - | 0.636 | .533 | - | - | - | - |
| DCI | Negative Dyadic Coping By Partner | 0.496 | .814 | 0.71 | .158 | - | - | 0.455 | .896 | - | - | - | - |
| DCI | Supportive Dyadic Coping By Oneself | 0.57 | .671 | 0.392 | .777 | - | - | 0.72 | .276 | - | - | - | - |
| DCI | Supportive Dyadic Coping By Partner | 0.571 | .671 | 0.355 | .853 | - | - | 0.453 | .943 | - | - | - | - |
| DS | Authority | - | - | 0.226 | .993 | - | - | - | - | - | - | - | - |
| DS | Disparagement | - | - | 0.399 | .759 | - | - | - | - | - | - | - | - |
| DS | Restrictiveness | - | - | 0.173 | .999 | - | - | - | - | - | - | - | - |
| EAQ | Degradation | 0.49 | .87 | 0.352 | .86 | - | - | 0.628 | .095 | - | - | - | - |
| EAQ | Isolation | 0.424 | .988 | 0.339 | .907 | - | - | 0.444 | .986 | - | - | - | - |
| EAQ | Property Damage | - | - | 0.188 | .998 | - | - | - | - | - | - | - | - |
| EAQ | Sexual Abuse | 0.449 | .93 | 0.481 | .575 | - | - | 0.754 | .104 | - | - | - | - |
| ECR | Anxiety | 0.985 | .001*** | - | - | - | - | - | - | - | - | - | - |
| ECR | Avoid | 0.926 | .001*** | - | - | - | - | - | - | - | - | - | - |
| ERC | Emotional Regulation | - | - | 0.404 | .744 | - | - | - | - | - | - | - | - |
| ERC | Lability Negativity | - | - | 0.503 | .41 | - | - | - | - | - | - | - | - |
| FDHI | Negative Involvement | 0.505 | .826 | 0.418 | .658 | - | - | 0.621 | .188 | - | - | - | - |
| FDHI | Positive Involvement | 0.49 | .885 | 0.551 | .206 | - | - | 0.582 | .386 | - | - | - | - |
| FDHI | Time Energy Involvement | 0.618 | .32 | 0.434 | .554 | - | - | 0.535 | .66 | - | - | - | - |
| MB | Marital Burnout | 0.385 | .999 | 0.212 | .995 | - | - | 0.596 | .446 | - | - | - | - |
| MDJS | Behavioral Jealousy | 0.405 | .989 | 0.562 | .32 | - | - | 0.635 | .378 | - | - | - | - |
| MDJS | Cognitive Jealousy | 0.426 | .959 | 0.342 | .87 | - | - | 0.647 | .342 | - | - | - | - |
| MDJS | Emotional Jealousy | 0.469 | .899 | 0.466 | .619 | - | - | 0.6 | .541 | - | - | - | - |
| OQ | Interpersonal Relations | 0.767 | .061 | 0.55 | .301 | 0.911 | .339 | 0.587 | .502 | 0.887 | .625 | 0.92 | .326 |
| OQ | Risk | 0.577 | .67 | 0.224 | .979 | 0.855 | .543 | 0.713 | .285 | 0.877 | .483 | 0.878 | .444 |
| OQ | Social Role | 0.576 | .604 | 0.389 | .778 | 0.87 | .626 | 0.489 | .885 | 0.884 | .634 | 0.876 | .63 |
| OQ | Symptom Distress | 0.691 | .057 | 0.388 | .734 | 0.885 | .477 | 0.645 | .1 | 0.904 | .441 | 0.895 | .431 |
| POWER | Partner Power | - | - | 0.437 | .695 | - | - | - | - | - | - | - | - |
| POWER | Self-Power | - | - | 0.353 | .833 | - | - | - | - | - | - | - | - |
| RQ | Relationship Questionnaire | - | - | 0.589 | .353 | - | - | - | - | - | - | - | - |
| SBNR | Secure Based Narrative Representational | - | - | 0.272 | .925 | - | - | - | - | - | - | - | - |
| SF | General Health | 0.528 | .801 | 0.523 | .505 | - | - | 0.816 | .099 | - | - | - | - |
| SF | Mental Health | 0.674 | .401 | 0.379 | .81 | - | - | 0.51 | .827 | - | - | - | - |
| SRES | Egalitarian | - | - | 0.462 | .462 | - | - | - | - | - | - | - | - |
| TCS | Anxiety | 0.481 | .876 | 0.707 | .068 | - | - | 0.629 | .362 | - | - | - | - |
| TCS | Depression | 0.528 | .769 | 0.376 | .807 | - | - | 0.606 | .514 | - | - | - | - |
| TCS | Dissociation | 0.482 | .886 | 0.649 | .198 | - | - | 0.579 | .675 | - | - | - | - |
| TCS | Sati | 0.402 | .976 | 0.561 | .383 | - | - | 0.52 | .82 | - | - | - | - |
| TCS | Sexual Problems | 0.462 | .903 | 0.475 | .563 | - | - | 0.5 | .875 | - | - | - | - |
| TCS | Sleep Dis | 0.388 | .989 | 0.465 | .644 | - | - | 0.585 | .616 | - | - | - | - |
